# Negative urgency partially accounts for the relationship between major depressive disorder and marijuana problems

**DOI:** 10.1186/s40479-018-0087-7

**Published:** 2018-05-16

**Authors:** Rachel L. Gunn, Kristina M. Jackson, Brian Borsari, Jane Metrik

**Affiliations:** 1 Center for Alcohol and Addiction Studies, Brown University School of Public Health, Providence, RI 02903 USA; 2 San Francisco VA Medical Center, San Francisco, California 94121 USA; 3 University of California, San Francisco, 94143 USA; 4 Providence VA Medical Center, Providence, RI 02908 USA

**Keywords:** Marijuana problems, Cannabis, Negative urgency, Depression, Major depressive disorder, UPPS-P

## Abstract

**Background:**

To goal of this study was to better understand mechanisms underlying associations between Major Depressive Disorder (MDD) and marijuana use and problems. Specifically, it was hypothesized that negative urgency (NU), the tendency to act rashly while experiencing negative mood states, would uniquely (compared to other impulsivity traits: positive urgency, sensation seeking, premeditation, and perseverance) account for the relationship between MDD and marijuana use and problems.

**Methods:**

Data were collected from a sample (*N* = 357) of veterans (*M* age = 33.63) recruited from a Veterans Affairs hospital who used marijuana at least once in their lifetime. Participants completed the SCID-NP to assess MDD, a marijuana problems scale, a Time-Line Follow-back to assess six-month marijuana use, and the UPPS-P Impulsive Behavior Scale for impulsivity.

**Results:**

Path analysis was conducted using bootstrapped (*k* = 20,000) and bias-corrected 95% confidence intervals (CIs) to estimate mediation (indirect) effects, controlling for age, sex, and race. Analyses revealed a significant direct effect of MDD on NU and NU on marijuana problems. Regarding mediational analyses, there was a significant indirect effect of MDD on marijuana problems via NU. The direct effect of MDD on marijuana problems was reduced, but remained significant, suggesting partial mediation. No other impulsivity scales accounted for the relationship between MDD and marijuana problems. In predicting marijuana use, there were no significant indirect effects for any impulsivity traits, including NU, despite significant bivariate associations between use and NU and MDD.

**Conclusions:**

Results suggest that high levels of NU may partially explain associations between MDD and marijuana problems, but not marijuana use. No other facets of impulsivity accounted for the relationship between MDD and marijuana use or problems, underscoring the specificity of NU as a putative mechanism and the importance of assessing NU in treatment settings.

## Background

Marijuana is the most commonly used illicit drug world-wide [1], with the majority of US states having legalized it for either recreational and/or medicinal use within the past decade. In the wake of these rapid social and legal changes, epidemiological research reveals that past-year cannabis use disorder (CUD) rates have increased in the general population [2] and have also more than doubled in the past decade among military veterans [3]. Among individuals with CUD (and other substance use disorders), rates of comorbid mood disorders are higher relative to those without CUD [2, 4, 5]. Comorbidity between mood disorders and SUDs including CUD is particularly common in veterans [6, 7], particularly post-deployment [8], calling for more research investigating potential mechanisms to explain this comorbidity.

### Major depressive disorder and marijuana use

Major Depressive Disorder (MDD) is one psychiatric disorder shown to be strongly associated with both CUD and marijuana problems in general populations [4, 9–13] and among veterans [5, 14]. Affective-motivational theory emphasizes the central role of negative affect in motivating drug use, including marijuana use specifically [15, 16]. Recent cross-sectional data suggest that marijuana users who experience MDD are more likely to have CUD than marijuana users without MDD [12]. Cross-sectional between-subject [5, 17] and prospective within-subject [18] empirical research in support of this theory suggests that greater intensity of negative affect associated with MDD leads to increased marijuana use to in order to cope with negative emotions. Yet, coping-oriented use of substances has also been shown to worsen affective symptoms of depression and to increase substance misuse [19, 20].

Evidence for the directionality of the association between MDD and CUD is mixed. Some longitudinal studies have provided evidence that cannabis use predicted increased symptoms of depression; whereas depressive symptoms did not predict increased cannabis use [21, 22]. However, this directionality was only found among adolescent girls in one study, limiting generalizability [22]. One meta-analysis of longitudinal studies found that heavy cannabis use may be associated with increased depressive symptoms, but did not explore the opposite direction (depressive symptoms to CUD [23]). In contrast, large epidemiological studies have also revealed MDD was prospectively associated with CUD and contributed to its etiology [24, 25]. Additional longitudinal work has suggested a bidirectional relationship between depressive symptoms and cannabis use from adolescence to young adulthood across five years of assessment in men [26].

### Impulsivity and marijuana use and problems

Impulsive personality traits have long been a hallmark characteristic for substance misuse and substance use disorders in general [27–29]. Certain facets of impulsivity, such as delay discounting, have been associated with greater marijuana use [30] and marijuana dependence [31]. Composite scores of attentional, motor, and nonplanning impulsivity have also been associated with marijuana problems [32, 33]. Importantly, the UPPS-P Impulsive Behaviors Scale [34] classifies impulsivity as multi-faceted construct [35, 36], in which certain traits are uniquely related to specific risky behaviors [37–40]. Each of these five impulsivity-like traits (negative urgency, positive urgency, sensation seeking, lack of premeditation, and lack of perseverance) have been found to be associated with marijuana use and related consequences [15, 41–43].

### Impulsivity, major depressive disorder, and marijuana use and problems

Impulsive personality traits may partially explain the association between MDD and marijuana use and problems. Specifically, negative urgency (NU), one facet of impulsivity characterized by rash action when experiencing emotional distress [36], may be of particular relevance to this comorbidity. When considering all facets of the UPPS-P model, NU and lack of perseverance specifically have been shown to relate to symptoms of MDD [44, 45]. NU has also been associated with marijuana use and problems in general populations [42, 46]. Relatedly, NU has been associated with alcohol use problems, particularly among those with higher levels of MDD [44, 47]. It may be that MDD places individuals at risk for marijuana problems via a similar mechanism. Thus, marijuana users with MDD may be more likely to act without thinking when upset or distressed. This in turn may lead to heavier use and a greater number of negative consequences related to marijuana use.

### The present study

In order to clarify the mechanisms linking MDD and problematic marijuana use, this study sought to examine whether NU would uniquely (compared to other impulsivity traits) explain the relationship between MDD and marijuana use and problems. Two specific questions are examined: 1) The extent to which higher NU accounts for the relationship between MDD and marijuana use and problems; and 2) Whether this effect is unique to NU, or if other impulsive personality traits also partially account for the relationship between MDD and marijuana use and problems.

## Methods

### Sample and procedure

Data were drawn from a larger prospective study examining marijuana use and affective disorders in returning Operation Enduring Freedom, Operation Iraqi Freedom, and Operation New Dawn (OEF/OIF/OND) veterans who were deployed post 9/11/2001 and who used marijuana at least once in his/her lifetime. Participants were recruited from a VHA facility in the Northeast US by utilizing the VHA OEF/OIF/OND Roster, an accruing database of combat veterans who have recently returned from military service in Iraq and Afghanistan and enrolled in VHA (see Metrik et al., 2016, for details of recruitment procedures). Veterans were screened for eligibility by telephone and were invited for a baseline visit, at which time they signed informed consent and completed a battery of interview and self-report assessments (including all measures analyzed in the current study). The study was approved by the university and local VHA Institutional Review Boards. Participants were compensated $50 upon completion of the study session. The original sample included 361 participants, from which four subjects were removed for missing data, resulting in a final *N* = 357.

### Measures

#### Structured clinical interview for DSM, non-patient edition (SCID-NP)

Was used to determine DSM-5 [48] diagnosis of current (past month) Major Depressive Disorder [49]. All SCID interviews were administered by research assistants, who were trained by the PI and required to demonstrate adherence and competence to the interview. All SCIDs were audiotaped and a random selection of the recordings (*n* = 72, 20%) were later rated by an independent doctorate-level clinician, resulting in excellent inter-rater reliability (ICC = .98–.99, 95%, CIs [.96–.99]). Any discrepancies were resolved in discussion with the PIs (BB and JM).

#### Marijuana problems

Marijuana-related problems were assessed with the Marijuana Problems Scale (MPS; [50]), a self-report 22-item questionnaire that evaluates problems experienced in the past 90 days related to marijuana use. A total count of combined minor and serious problems was used rather than a severity score. The MPS has strong internal consistency in previous studies [50, 51] and in this sample (α = .91).

#### Marijuana use

The Time-Line Follow-Back Interview (TLFB; [52, 53]) was used to record percent days of marijuana use over the six months prior to the visit.

#### Impulsive personality traits

Facets of impulsivity were assessed using the Short UPPS-P Impulsive Behavior Scale [54]. The UPPS-P is a 20-item self-report inventory which uses a 4-point likert scale to assesses five subscales of impulsive personality (negative urgency [NU], positive urgency [PU], sensation seeking [SS], lack of premeditation [PM], and lack of perseverance [PS]), each demonstrating high levels of internal consistency in previous studies [54]. These subscales demonstrated acceptable (PS α = .69, SS α = .62) to good (NU α = .77, PU α = .83, PM α = .82) internal consistency.

### Data analytic strategy

Descriptive statistics and bivariate (point biseral for dichotomous variables) correlations were first examined. Next, hypothesized mediational models were examined. MDD was specified as the predictor, or independent variable; marijuana use and problems were specified as the outcomes, and impulsivity measures were specified as the mediators of interest. Several studies show that the several facets of the UPPS-P model of impulsivity are highly intercorrelated [40, 55]. Including all five traits in a single model can create statistical suppression and make it difficult to interpret each unique effect [56]. In order to address this issue, we first examined the correlations between each trait to guide decisions for which traits to examine for mediation. Specifically, we examined Negative Urgency [NU], Positive Urgency [PU], and Lack of Perseverance [PS] because, as reported below, they were significantly associated with both MDD and marijuana outcomes in this sample. A total of eight mediational models were tested. First, we tested separate models for each of the marijuana outcomes (marijuana problems and percent marijuana use days from the TLFB), for each of the mediators (NU, PU, and PS), which resulted in a total of six models. Then, we tested two models with all mediators entered simultaneously in order to examine whether any significant associations remained. Finally, given the cross-sectional nature of the data, follow-up mediation analyses with reverse directionality were tested, where marijuana use and problems were specified as the predictor, or independent variable; MDD was specified as the outcome, with impulsivity measures remaining as mediators of interest.

The primary data analyses were a structural equation model (SEM) with maximum likelihood estimation to using AMOS 24.0 [57]. All models regressed the dependent variable (marijuana problems or use) onto covariates (age [continuous], sex [binary], and race [binary, non-Hispanic Caucasian]). Covariates were allowed to correlate with each other in single and multiple mediator models. In order to estimate mediation effects, bootstrapped (*k =* 20,000) and bias-corrected 95% confidence intervals (CIs) were estimated for the indirect effects [58]. Mediation is tested by examining the direct, indirect, and total effects. Significant mediation effects are apparent when indirect effects are significant and total effects are reduced in the presence of the mediator. To assess the degree to which the structural models fit the sample variance-covariance data, two criteria of model fit were relied upon: the Comparative Fit Index (CFI: [59]), and the root-mean- square error of residual approximation (RMSEA: [60]). Although guidelines for good fit vary, values above .90 for CFI and below .05 for RMSEA are considered acceptable [61].

## Results

### Descriptive statistics and bivariate correlations

Table 1 presents sample demographics and substance use descriptive statistics. Table 2 presents bivariate correlations. As expected, MDD was positively associated with marijuana use and marijuana problems. Also as expected, MDD was positively associated with NU. Additionally, MDD was associated with PU, and PS, and PM. In this sample, marijuana problems were positively associated with NU, PU, and PS. Marijuana use was also associated with NU, PU, PS, as well as PM.

**Table 1:** Descriptive Statistics

Variable	*n*	%
Sex (Male)	335	94
Race		
White	286	80
Black/African American	16	4
Asian	6	2
Native Hawaiian/Pacific Islander	2	01
American Indian/Alaska Native	2	01
Multiracial/Other	25	8
Ethnicity		
Hispanic/Latino(a)	88	25
Marital Status		
Single/Never Married	115	32
Married/Living with Partner	170	48
Divorced/Separated	72	20
Employment Status		
Employed	279	78
Unemployed	123	34
Student	27	8
Military service	100	28
DSM-5 Diagnoses		
Cannabis Use Disorder, current	52	15
Cannabis Use Disorder, lifetime	132	37
Major Depressive Disorder	53	15
	*M*	*SD*
Age	33.63	9.46
Years of Education	13.72	2.12

*N* = 357; Employment Status: participants were instructed to ‘check all that apply’ regarding their employment status over the past year

**Table 2:** Bivariate Correlations

Variable	1.	2.	3.	4.	5.	6.	7.	8.	9.	10.	11.	M (sd)
1. MJ problems	–	.37***	.14**	.21***	.05	.13*	.02	.21***	−.10	.01	−.10	1.07 (2.71)
2. MJ use		–	.12*	.17**	.04	.13*	.11*	.26***	−.18**	.07	−.01	16.13 (32.7)
3. PU			–	.56***	.19***	.15**	.42***	.19***	−.16**	−.14**	−.10	2.16 (.72)
4. NU				–	−.01	.21***	.43***	.29***	−.08	.01	.01	1.75 (.67)
5. SS					–	−.10	.07	−.07	−.21**	−.19**	−.00	2.88 (.69)
6. PS						–	.41***	.16**	−.08	.05	−.05	1.61 (.49)
7. PM							–	.23***	−.14**	−.00	.03	1.76 (.56)
8. MDD								–	−.02	−.16**	.04	--^a^
9. Age									–	−.01	.08	--^a^
10. Sex										–	−.02	--^a^
11. Race											–	--^a^

MJ Marijuana problems, MJ use % Marijuana use days, *PU* positive urgency, *NU* negative urgency, *SS* sensation seeking, *PS* lack of perseverance, *PM* lack of premeditation, *MDD* Major Depressive Disorder. ***p < .001, **p < .01, *p < .05; ^a^ Mean and standard deviation not presented due to dichotomous variable, Table 1 presents appropriate descriptive statistics for these variables

### Mediation models

A set of mediation models for each of the two outcomes were first tested. We did not examine mediation by SS or PM due to lack of association with MDD (SS) and marijuana use (SS) and problems (SS and PM). Results are presented in Table 3, the top portion of which presents effects for single mediator models and the bottom portion for multiple mediator models.

**Table 3:** Results of Path models: Indirect and Direct effects of MDD on Marijuana Use and Problems, via Impulsive Personality Traits

	Direct path to mediator *(a path)*	Marijuana Use *(b path)*	Marijuana Problems *(b path)*
Mediator	*B* (*SE*)	*B* (*SE*)	*B* (*SE*)
Single Mediator Models			
Direct Effect			
NU	.29 (05)***	.09 (.05)	.15 (.06)*
PU	.21 (.06)***	.06 (.05)	.08 (.08)
PS	.15 (.06)**	.08 (.06)	.09 (.05)
Indirect Effect			
NU		.03 (.02)	.05 (.02)*
PU		.01 (.01)	.02 (.02)
PS		.01 (.01)	.01 (.01)
Multiple Mediator Model			
Direct Effect			
NU	.29 (.05)***	.07 (.06)	.15 (.07)*
PU	.19 (.06)***	.01 (.06)	−.01 (.07)
PS	.16 (.06)**	.06 (.06)	.07 (.06)
Indirect Effect (a x b)			
		.03 (.02)*	.05 (.02)*
Total Effect			
		.25 (.07)***	.22 (.07)***

*NU* negative urgency, *PU* positive urgency, *PS* lack of perseverance, *MDD* Major Depressive Disorder. Parameters are standardized. All models control for age, sex, and race. ***p < .001, **p < .01, *p < .05

#### Marijuana use

In predicting marijuana use frequency, single mediator models did not return significant indirect effects for NU, PU or PS, indicating they did not account for the association between MDD and marijuana use. In the multiple mediator model, there was a significant indirect effect of MDD on marijuana use (β = .03, *p* < .05, 95% CI [.01, .09]), however none of the proposed mediators accounted for this indirect effect (see Table 3). Age was the only variable with a significant direct effect on marijuana use (β = −.16, *p* < .001), while NU, PU, and PS remained non-significant (see bottom panel of Table 3). Results were consistent in the mediation model with reverse directionality, as there were no indirect effects of MDD on marijuana use.

#### Marijuana problems

**Fig. 1:**
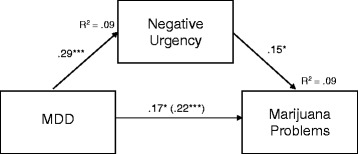
NU partially accounts for the association between MDD and Marijuana Problems *Note*. Parameter estimates are from the single mediator model. Mediational path model of the association between MDD, NU, and marijuana problems. Single directional arrows represent standard regression weights. The direct effect of MDD on marijuana problems before NU was included in the model is presented in parentheses. Models controlled for age, sex, and race. ***p < .001, **p < .01, *p < .05

In separate single mediator models examining the association between MDD and marijuana problems, there was a significant indirect effect of NU, as hypothesized (see top panel of Table 3). This model suggested that NU significantly accounted for the relationship between MDD and marijuana problems (see Fig. 1). As can be seen in Fig. 1, a significant direct effect of MDD on marijuana problems remained when NU was in the model, suggesting partial mediation. As can also be seen in the model, there was a significant direct effect of MDD on NU and NU on marijuana problems. This model fit adequately, *χ*^*2*^ (6) = 11.84, *p* = .07, CFI = .91, NFI = .85, RMSEA = .05. As expected, neither PU nor PS accounted for the relationship between MDD and marijuana problems in single mediator models (see top panel, Table 3). In the mediation model with reverse directionality, examining the association between marijuana problems and MDD, there were significant total effects (β = .22, *p* = .001), direct effects (β = .16, *p* = .01), and indirect effects (β = .05, *p* < .01, 95% CI [.02, .09]) of MDD on marijuana problems, providing comparable evidence for partial mediation.

In the multiple mediator model, the indirect effect of NU remained significant when PU and PS were still in the model, suggesting it significantly accounted for the association between MDD and marijuana problems (see bottom panel of Table 3). This model fit adequately, *χ*^*2*^ (12) = 38.53, *p* < .001, CFI = .88, NFI = .85, RMSEA = .08, and revealed significant total effects, direct effects, and indirect effects of MDD on marijuana problems providing evidence for partial mediation (see bottom panel of Table 3). Consistent with the single mediator model, the reverse multiple mediator model also found significant total effects (β = .22, *p* = .001), direct effects (β = .16, *p* = .01), and indirect effects (β = .06, *p* < .01, 95% CI [.02, .11]) of MDD on marijuana problems providing comparable evidence for partial mediation.

## Discussion

The goal of the present study was to better understand mechanisms associated with high rates of co-occurring MDD and problematic marijuana use by examining the role of specific facets of the UPPS-P model of impulsive personality [35, 36] in this comorbidity. To our knowledge, the current study is the first to systematically examine the role of these personality traits between MDD and marijuana use and problems. As hypothesized, we found that NU partially accounted for the relationship between MDD and marijuana problems, but this was not true of the other impulsivity traits.

Consistent with previous research [5, 9], we found that MDD was associated with marijuana use and problems. Although this is not the first study to examine the relationship between MDD and marijuana use and problems, it is the first to examine how individual dispositions to impulsive/rash action may help explain the association between these two clinical problems. We were also able to replicate previous research suggesting an association between MDD and NU [44, 45]. The current study expands this literature by suggesting that individuals with MDD and high levels of NU are in turn more likely to have greater number of marijuana problems. Importantly, our results also suggest that NU is the only trait in the UPPS model that accounted for the association between MDD and marijuana problems. This is consistent with theory suggesting the increased negative affect experienced by those with mood disorder, such as MDD, may lead to increased substance-related problems [15]. This high rate of negative affect may be particularly problematic for individuals also high in NU, who may in turn be more likely to act impulsively when experiencing negative mood states, and thus be more likely to experience problems related to substance use. Although results in support of this mediational pathway are compelling, remaining variance in our models suggest alternative pathways may exist to explain this comorbidity. For example, marijuana coping motives have also been shown to mediate the relationship between MDD or other affective vulnerabilities, such as anxiety and distress tolerance, and marijuana use and problems in general and veteran populations [5, 13, 62–64].

Contrary to our hypothesis, this mediational pathway was not present for marijuana use, indicating that NU is specifically implicated in the experience of problematic marijuana use. This is consistent with work suggesting that NU is a robust predictor of both marijuana problems [41, 65] and alcohol problems [66–68], although the relationship between NU and marijuana problems has received far less attention. Previous studies have used similar methods to explain the relationship between MDD and alcohol use and problems. In one study of young adult drinkers, NU significantly mediated the relationship between depressive symptoms and alcohol problems when controlling for alcohol use [47]. Similarly, King and colleagues [67] examined which of the UPPS-P model traits might moderate the relationship between depressive symptoms and alcohol problems among college student drinkers. They found that although NU was the strongest predictor of alcohol problems, lack of premeditation was the only moderator of depressive symptoms and alcohol problems. Although this study examined impulsivity traits as moderators, it is important to mention as they found unique associations between NU and depressive symptoms when examining alcohol problems, which is consistent with our findings with marijuana problems.

The present study expands this knowledge by not only showing that the relationship between MDD and marijuana problems may be partially explained by NU, but also in a population of military veterans. Veterans often have higher rates of MDD and substance use disorders including CUD compared to the general population [69, 70], and thus an important target population for intervention. The present research has important treatment and prevention implications for individuals with MDD and marijuana problems. Given the emerging evidence of an association between NU and marijuana problems in a number of different populations, it may be important for clinicians to assess for NU to be aware of the additional risk for those with MDD and high levels of NU. Although we focused on the directional pathway of MDD predicting marijuana-related behvaiors, it is also important to acknowledge that longitudinal evidence also exists to suggest that marijuana use is prospectively associated with depressive symptoms and other mood disorders [see review: 16]. Therefore, individuals at risk for depression and those with MDD should consider avoiding using marijuana, as it could in turn exacerbate the severity of depressive symptoms.

## Limitations and conclusions

A few limitations should be considered when interpreting the results of this study. First, our data are cross-sectional and cannot provide a test of the model that MDD leads to higher levels of NU, and in turn increased marijuana problems. It is possible that greater predisposition to NU precedes the development of both MDD and marijuana problems. There also appears to be support for bi-directionality in the effects, such that marijuana problems and use could also lead to or exacerbate symptoms of MDD. In fact, we tested both directional pathways with the mediation analyses and found consistent results, suggesting this is likely a bidirectional relationship, and that NU may be a consistent mediator for both pathways to comorbidity. Future prospective modeling is needed to directly empirically evaluate the extent to which depression may further maintain problematic patterns of marijuana use and to clarify the role of NU. Second, the timeframes by which the measures are assessed vary. MDD was assessed over the past month, and marijuana use and problems over longer timeframes (six and three months, respectively), further limiting any conclusions about directionality. However, regardless of the inability of the present study to resolve directionality, we believe it still sheds important light on the mechanisms linking MDD and problematic marijuana use.

Third, the use of a veteran population meant there was a very small number of women in the sample, (although the proportion in this study was representative of the 5–10% of women among U.S. military veterans). This limits the generalizability to women in non-veteran populations. Fourth, the use of frequency over quantity of marijuana use in the TLFB may have reduced the likelihood of finding an association with NU, as quantity may me a more sensitive test of problematic use compared to frequency. Additionally, relevant to the TLFB, is the length of the assessment window (6 months), which may be increasingly subject to retrospective recall bias when compared to shorter time periods. Although research suggests that TLFB reports underestimate frequency and quantity, this recall is not temporally biased. In other words, reports do not change significantly across time period (from 30 to 60 to 366 days [71]; 30 and 180-day intervals [72]).

Despite these limitations, the findings presented here provide important information about the risk for problematic marijuana use among individuals with co-occurring depressive symptoms, and the role of high levels of NU. Moreover, they suggest that relative to other common impulsive personality traits, individuals high in NU are at particular risk for problems related to their marijuana use. Future studies would benefit from studying these associations longitudinally and with a more diverse sample of both men and women in order to determine possible causality between MDD, NU, and problematic marijuana use.

## Abbreviations

MDD: Major Depressive Disorder
NU: negative urgency
PM: lack of premeditation
PS: lack of perseverance
PU: positive urgency
SS: sensation seeking
